# A2M is a potential core gene in intrahepatic cholangiocarcinoma

**DOI:** 10.1186/s12885-021-09070-2

**Published:** 2022-01-03

**Authors:** Guanran Zhang, Xuyue Liu, Zhengyang Sun, Xiaoning Feng, Haiyan Wang, Jing Hao, Xiaoli Zhang

**Affiliations:** 1 Key Laboratory for Experimental Teratology of Ministry of Education, Department of Histology & Embryology, School of Basic Medical Sciences, Shandong University, Jinan, 250012 Shandong China; 2 School of Information Engineering, Zhengzhou University, Zhengzhou, 450001 Henan China; 3 Shandong Provincial Hospital Affiliated to Shandong First Medical University, Jinan, China

**Keywords:** Intrahepatic cholangiocarcinoma, Hub genes, Gene expression profiling, A2M

## Abstract

**Background:**

Intrahepatic cholangiocarcinoma (ICC) is a type of malignant tumor ranking the second in the incidence of primary liver cancer following hepatocellular carcinoma. Both the morbidity and mortality have been increasing in recent years. Small duct type of ICC has potential therapeutic targets. But overall, the prognosis of patients with ICC is usually very poor.

**Methods:**

To search latent therapeutic targets for ICC, we programmatically selected the five most suitable microarray datasets. Then, we made an analysis of these microarray datasets (GSE26566, GSE31370, GSE32958, GSE45001 and GSE76311) collected from the Gene Expression Omnibus (GEO) database. The GEO2R tool was effective to find out differentially expressed genes (DEGs) between ICC and normal tissue. Gene Ontology (GO) function and Kyoto Encyclopedia of Genes and Genomes (KEGG) pathway enrichment analysis were executed using the Database for Annotation, Visualization and Integrated Discovery (DAVID) v 6.8. The Search Tool for the Retrieval of Interacting Genes (STRING) database was used to analyze protein–protein interaction of these DEGs and protein–protein interaction of these DEGs was modified by Cytoscape3.8.2. Survival analysis was performed using Gene Expression Profiling Interactive Analysis (GEPIA) online analysis tool.

**Results:**

A total of 28 upregulated DEGs and 118 downregulated DEGs were screened out. Then twenty hub genes were selected according to the connectivity degree. The survival analysis results showed that A2M was closely related to the pathogenesis and prognosis of ICC and was a potential therapeutic target for ICC.

**Conclusions:**

According to our study, low A2M expression in ICC compared to normal bile duct tissue was an adverse prognostic factor in ICC patients. The value of A2M in the treatment of ICC needs to be further studied.

## Introduction

Intrahepatic cholangiocarcinoma (ICC) is defined as a type of malignant tumor originating from epithelium of secondary bile duct and its branches [1–3]. ICC is the second most familiar primary liver cancer with increasing incidence [4, 5]. Median overall survival (OS) for ICC patients is 12 to 18 months, with 5-year OS rates of less than 5% [6–8]. Surgical excision plus adjuvant therapy is the main treatment methods at present, but only 15% of patients are qualified for surgery [9] due to its difficulty in detection. MsMab-1 that is an effective antibody against isocitrate dehydrogenase 1/2 (IDH1/2) mutation may be a therapeutic target for small duct type of ICC, but other subtypes still lack therapeutic targets [10]. Local treatments such as thermoablation, stereotactic radiotherapy and chemotherapy might prolong the survival time and improve the quality of life for some patients, but the overall prognosis is poor. Until now, ICC remains difficult to be cured and remains to be urgent to explore new therapeutic targets of ICC.

In this research, we attempted to discover original prognostic index for ICC patients and struggled for supplying potential therapeutic targets. We analyzed the gene expression profiling data from the Gene Expression Omnibus (GEO) database by bioinformatics technique to dig out the DEGs between normal human tissue and ICC. Then, we performed Gene Ontology function and Kyoto Encyclopedia of Genes and Genomes pathway enrichment analysis of DEGs. After that, a protein–protein interaction (PPI) network was built on The Search Tool for the Retrieval of Interacting Networks Genes (STRING) database and hub genes of ICC were screened out by cytoscape (3.8.2). DAVID tools were used to make the functional analyses of hub genes. We performed survival analysis of these hub genes using the online tool Gene Expression Profiling Interactive Analysis (GEPIA). Finally, A2M was screened out. All in all, the purpose of this study was to discover the biomarkers that was used for diagnosis, clinical treatment, and monitoring disease progression by analyzing the gene changes that took place during disease progression to improve the comprehension of the mechanism of ICC.

## Materials and methods

### Data source

The gene expression datasets in this research were obtained from the GEO database (https://www.ncbi.nlm.nih.gov/geo/). All of the datasets were freely downloaded, and no experiments on humans or animals were done by any of the authors.

### Algorithm idea of judge function

We used Excel to filter the data obtained from GEO database, and used Padj < 0.05 and |logFC| ≥ 1.0 as the standard to obtain DEGs. Then we exported them as text files, so that each line contained only one gene symbol, which were standardized texts.

**Fig. 1:**
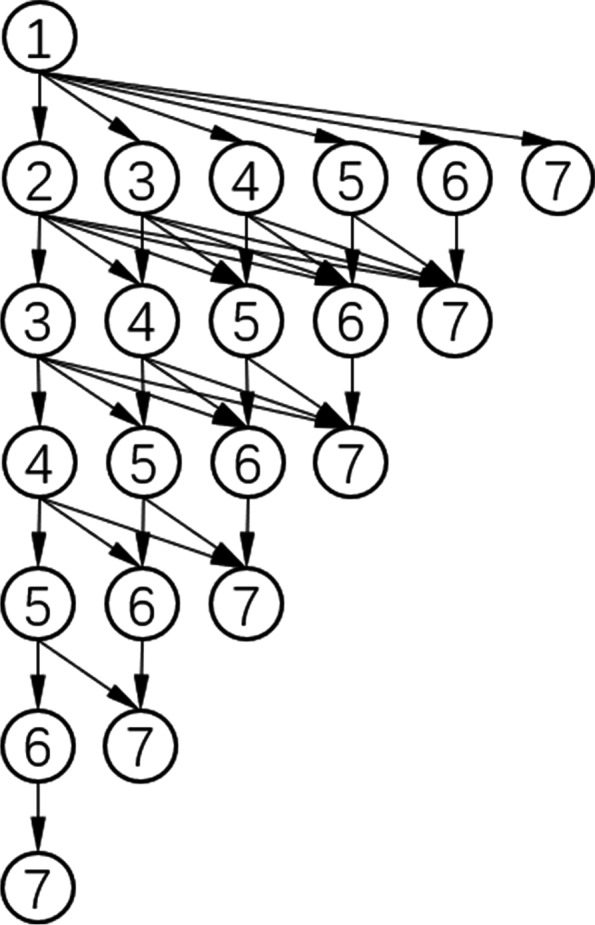
The algorithm idea of judge function

Digital nodes 1-7 represent 7 standardized texts. We start from any node and proceed along the arrow. We can only advance on the next line, but cannot advance within the same line or return to the previous line. All nodes on the path are all the normalized texts selected by the judge function (Fig. 1).

### Comparison program steps

**Fig. 2:**
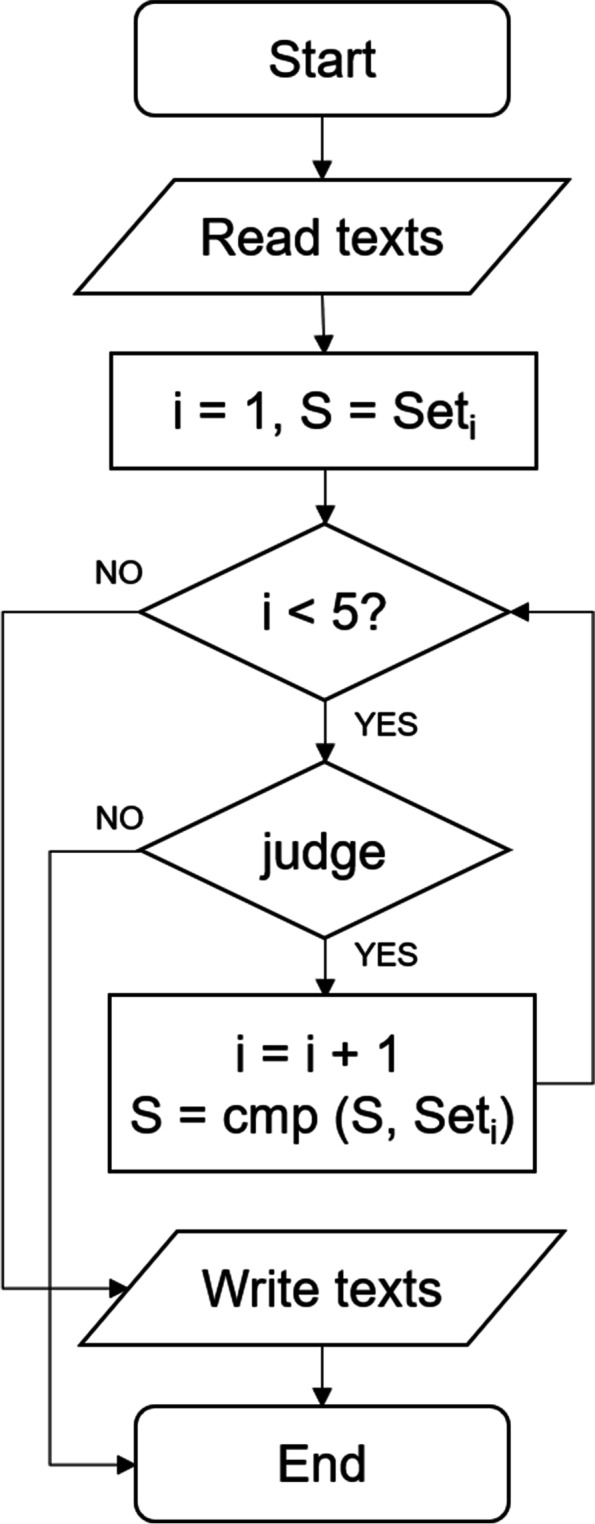
The steps of comparison program. S is the current comparison result and represents the same DEGs of the normalized texts that the judge function have passed through. Set represents normalized texts. I is cyclic variable, which represents the number of times the program loops

First of all, the comparison program read the standardized texts and determined whether the cyclic variable i was less than 5. If i was less than 5, we used the judge function to select the next dataset, and then used the cmp function to compare the DGEs of the standardized texts, and at the same time i value plus 1. Circulate according to the above method until i becomes 5, and when i was no longer less than 5, the comparison program output the results (Fig. 2). In addition, when the length of the path generated by the judge function was too short without 5 nodes, the path was invalid, the program ended, and the results were not produced. In conclusion, the results generated by comparison program contained gene symbol of common DEGs.

### Data selection

We found 2491 series about human ICC from the database. After careful selection, we screened out seven gene expression profiles (GSE26566, GSE31370, GSE32958, GSE33327, GSE89749, GSE45001 and GSE76311) that met the requirements. Then, we designed a program named comparison to process the seven gene expression datasets. The optimal results were obtained when five of the seven gene expression profiles were selected, and the highest number of differentially expressed genes (DEGs) was 146. Finally, five gene expression profiles (GSE26566, GSE31370, GSE32958, GSE45001 and GSE76311) were selected based on the number of differentially expressed genes (DEGs). Among them, GSE26566 was based on GPL6104 Platform (Illumina humanRef-8 v2.0 expression beadchip), GSE31370 was based on GPL10558 Platform (Illumina HumanHT-12 V4.0 expression beadchip), GSE32958 was based on GPL6244 Platform ([HuGene-1_0-st] Affymetrix Human Gene 1.0 ST Array [transcript (gene) version]), GSE45001 was based on GPL14550 Platform (Agilent-028004 SurePrint G3 Human GE 8x60K Microarray) and GSE76311 was based on GPL17586 Platform ([HTA-2_0] Affymetrix Human Transcriptome Array 2.0 [transcript (gene) version]).

### Data processing of DEGs

We used the GEO2R analysis tool (https://www.ncbi.nlm.nih.gov/geo/geo2r/) to discover the DEGs between ICC and healthy samples and screen out the DEGs based on the value of adjusted *P*-value (Padj) and fold change (|logFC|). The cross-platform normalization was performed by normalizeBetweenArrays of limma package in Bioconductor. The genes with Padj < 0.05 and |logFC| ≥ 1.0 were selected as DEGs. The DEGs with log FC < − 1.0 were defined as downregulated genes, while the DEGs with log FC > 1.0 were defined as upregulated genes. Then we selected the common part of DEGs by drawing the Venn diagrams using the webtool (bioinformatics.psb.ugent.be/webtools/Venn/).

### GO and KEGG pathway analysis of DEGs

The results of large dimensions functional enrichment research that gene function is divided into biological process (BP), cellular component (CC) and molecular function (MF) are often obtained by GO analysis. KEGG is a database resource for understanding advanced functions and biological systems from molecular level information, particularly genomic sequencing generated from large molecular datasets. KEGG is one of the most commonly used databases of biological information in the world. We used Database for Annotation, Visualization and Integrated Discovery (DAVID) v6.8 that was an online bioinformatics database with biological data and analytical tools, providing users with gene and protein functional annotation information to access biological information to get the results of GO analysis and KEGG pathway enrichment analysis of DEGs in this research (https://david.ncifcrf.gov/) [11]. The data that false discovery rate (FDR) < 0.01 and gene counts ≥10 were taken as statistically meaningful.

### PPI network construction and hub genes identification

The Search Tool for the Retrieval of Interacting Networks Genes (STRING) database (http://string-db.org/) was used for the PPI networks functional enrichment analysis. The DEGs obtained previously were entered into the STRING database to explore possible PPI relationships. The minimum required interaction score of PPI pairs was set as 0.400. After that, we used cytoscape software to modify the PPI network (www.cytoscape.org/). We used the plugin cytoHubba of cytoscape software to compute the connectivity degree of each node. Protein nodes with higher degree were more important in the whole PPI network. The top 20 genes with the highest connectivity degree were defined as hub genes. After that, we made the GO and KEGG pathway analysis of the selected hub genes using DAVID online analytical tools. The data of upregulated genes with FDR < 0.01 and gene counts ≥5 were taken as statistically meaningful and the data of downregulated genes with FDR < 0.01 and gene counts ≥10 were taken as statistically meaningful.

### Survival analysis of hub genes

The Gene Expression Profiling Interactive Analysis (GEPIA) is a TCGA visualization tool developed by Zhang’s Lab of Peking University (http://gepia.cancer-pku.cn/index.html). We used GEPIA to perform the survival analysis of ICC patients. ICC patients were divided into low group and high group according to the best cutoff values of mRNA expression calculated manually. *P < 0.05* was considered to be statistically significant for disease free survival analysis and overall survival analysis.

## Results

### Selection of datasets

**Fig. 3:**
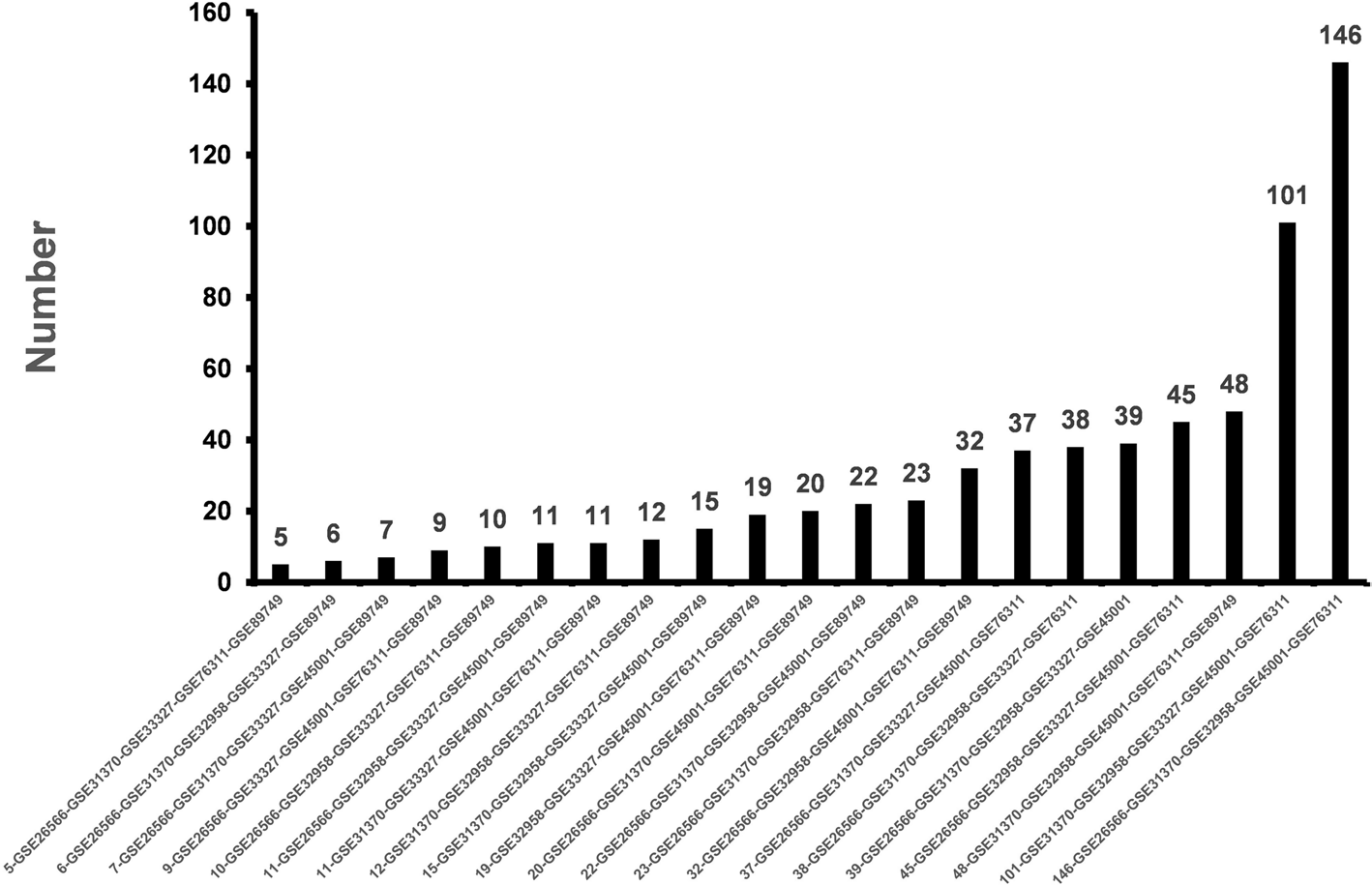
The number of common DEGs about 21 combinations

According to the type, number and year of cases, we artificially selected seven gene expression profiles (GSE26566, GSE31370, GSE32958, GSE33327, GSE89749, GSE45001 and GSE76311) that met the requirements from 2491 series about human ICC. Five gene expression profiles were screened out from the 7 gene expression profiles with a total of 21 combinations. After that, the comparison program we designed used the backtracking algorithm to traverse the 21 combinations when five gene expression profiles were selected from seven gene expression profiles, and listed the common DEGs of each combination. Then we showed the number of common DEGs that appeared in each combination (Fig. 3).

### Identification of DEGs

**Fig. 4:**
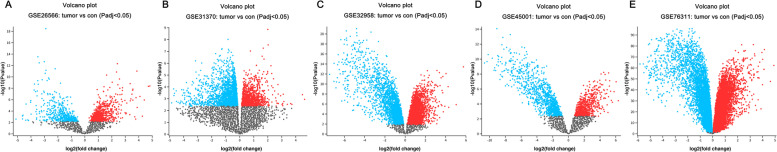
Differential expression of genes. **A** GSE26566 data, **B** GSE31370data, **C** GSE32958data, **D** GSE45001data, **E** GSE76311 data. The red dots refer to upregulated genes that were selected based on logFC > 0 and Padj < 0.05, the blue dots refers to downregulated genes that were selected based on logFC < 0 and Padj < 0.05, and the black dots mean genes change without significant difference

**Fig. 5:**
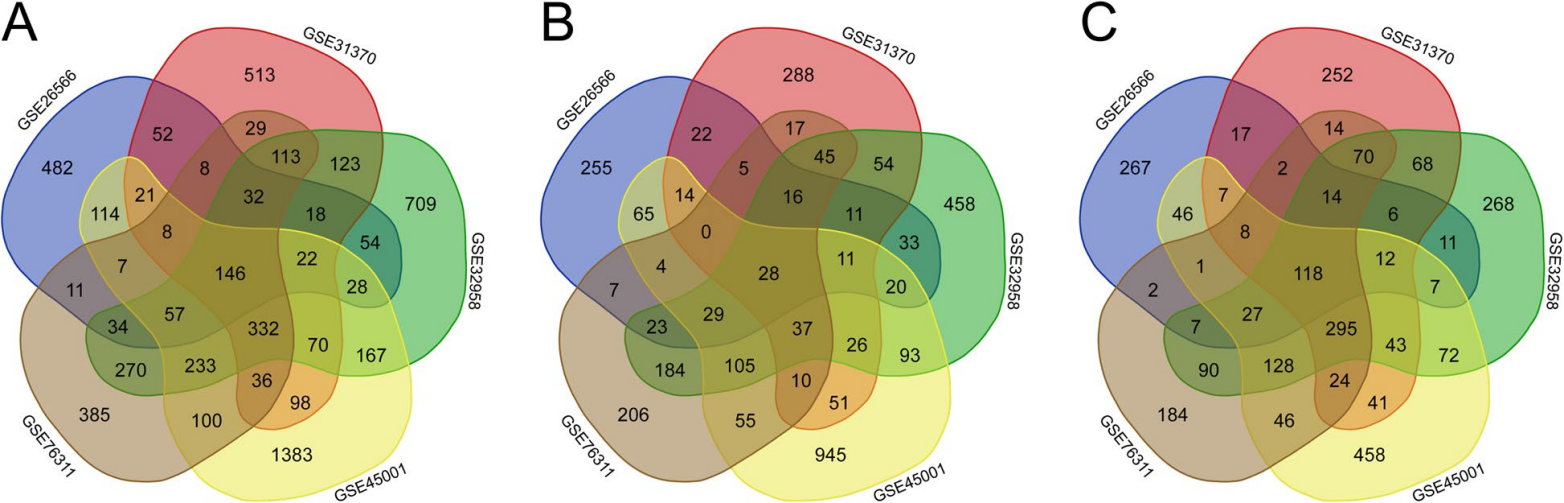
Venn diagram of DEGs in five databases. **A** Differentially expressed genes. **B** Upregulated genes. **C** Downregulated genes

We used the comparison program to pick out five microarray datasets (GSE26566, GSE31370, GSE32958, GSE45001 and GSE76311) and made the analysis in this study. Among them, GSE26566 included 104 ICC samples and 6 normal samples, GSE31370 contained 6 ICC samples and 5 normal samples, GSE32958 comprised 16 ICC samples and 7 normal samples, GSE45001 involved 10 ICC samples and 10 normal samples and GSE76311 contained 92 ICC samples and 93 normal samples (Table 1). We obtained DEGS by comparing the gene expression level in ICC samples and normal samples. One thousand ninety-three DEGs that contained 542 upregulated genes and 551 downregulated genes were screened out from GSE26566 using *P* < 0.05 and |logFC| ≥ 1.0 as the selection criteria. One thousand six hundred twenty-four DEGs that involved 634 upregulated genes and 990 downregulated genes were picked out from GSE31370. From GSE32958, 2407 DEGs comprising 1172 upregulated genes and 1235 downregulated genes were identified. Two thousand eight hundred twenty-four DEGs were found from GSE45001, including 1492 upregulated genes and 1332 downregulated genes. And 1801 DEGs containing 771 upregulated genes and 1030 downregulated genes were screened out from GSE76311. Figure 4 shows the differential expression of genes in each of the five microarrays. Then, we drew the venn diagram to obtain the intersection of the DEGs in five databases (Fig. 5). Ultimately, we got 146 DEGs containing 28 upregulated genes and 118 downregulated genes of the selected five datasets.

**Table 1:** Information of the five gene datasets from the GEO database

Dataset ID	ICC	Normal	Total number
GSE26566	104	6	110
GSE31370	6	5	11
GSE32958	16	7	23
GSE45001	10	10	20
GSE76311	92	93	185

Abbreviations: *GEO* Gene Expression Omnibus, *ICC* intrahepatic cholangiocarcinoma

### Functional enrichment analyses of DEGs

The results of Gene Ontology function and Kyoto Encyclopedia of Genes and Genomes pathway enrichment analysis for DEGs were obtained using the DAVID v6.8 (Tables 2 and 3). Gene Ontology includes Molecular Function (MF), biological process (BP) and cellular component (CC). From the results of GO analysis, we can draw a conclusion that the downregulated genes were principally concentrated in BPs and CCs, including oxidation-reduction process, negative regulation of endopeptidase activity, metabolic process, extracellular exosome, blood microparticle, extracellular space, extracellular region, mitochondrion and mitochondrial matrix. As for the molecular function, the downregulated genes were mainly related to the receptor binding. From the results of KEGG pathway enrichment analysis, we drew a conclusion that the downregulated genes were significantly concentrated in metabolic pathways, fatty acid degradation, biosynthesis of antibiotics, complement and coagulation cascades, peroxisome and carbon metabolism. As for the upregulated genes, they were principally concentrated in extracellular matrix organization, cell adhesion, ECM-receptor interaction, Focal adhesion and PI3K-Akt signaling pathway.

**Table 2:** Results of GO and KEGG pathways enrichment analysis of upregulated genes

Category	Term	Description	Count	FDR
BP term	GO:0030198	Extracellular matrix organization	9	1.80E-08
BP term	GO:0007155	Cell adhesion	11	1.80E-08
KEGG pathway	hsa04512	ECM-receptor interaction	6	5.77E-06
KEGG pathway	hsa04510	Focal adhesion	6	2.11E-04
KEGG pathway	hsa04151	PI3K-Akt signaling pathway	6	0.001699684

**Table 3:** Results of GO and KEGG pathways enrichment analysis of downregulated genes

Category	Term	Description	Count	FDR
BP term	GO:0055114	Oxidation-reduction process	23	3.10E-08
BP term	GO:0010951	Negative regulation of endopeptidase activity	10	4.37E-05
BP term	GO:0008152	Metabolic process	10	2.83E-04
CC term	GO:0070062	Extracellular exosome	62	1.23E-18
CC term	GO:0072562	Blood microparticle	17	1.20E-13
CC term	GO:0005739	Mitochondrion	28	1.51E-06
CC term	GO:0005615	Extracellular space	28	1.54E-06
CC term	GO:0005576	Extracellular region	28	4.39E-05
CC term	GO:0005759	Mitochondrial matrix	12	1.10E-04
MF term	GO:0005102	Receptor binding	14	1.94E-04
KEGG pathway	hsa01100	Metabolic pathways	44	1.04E-11
KEGG pathway	hsa00071	Fatty acid degradation	11	1.47E-09
KEGG pathway	hsa04610	Complement and coagulation cascades	11	1.78E-07
KEGG pathway	hsa01130	Biosynthesis of antibiotics	16	3.55E-07
KEGG pathway	hsa04146	Peroxisome	10	7.44E-06
KEGG pathway	hsa01200	Carbon metabolism	10	8.53E-05

### PPI network construction and hub genes identification

**Fig. 6:**
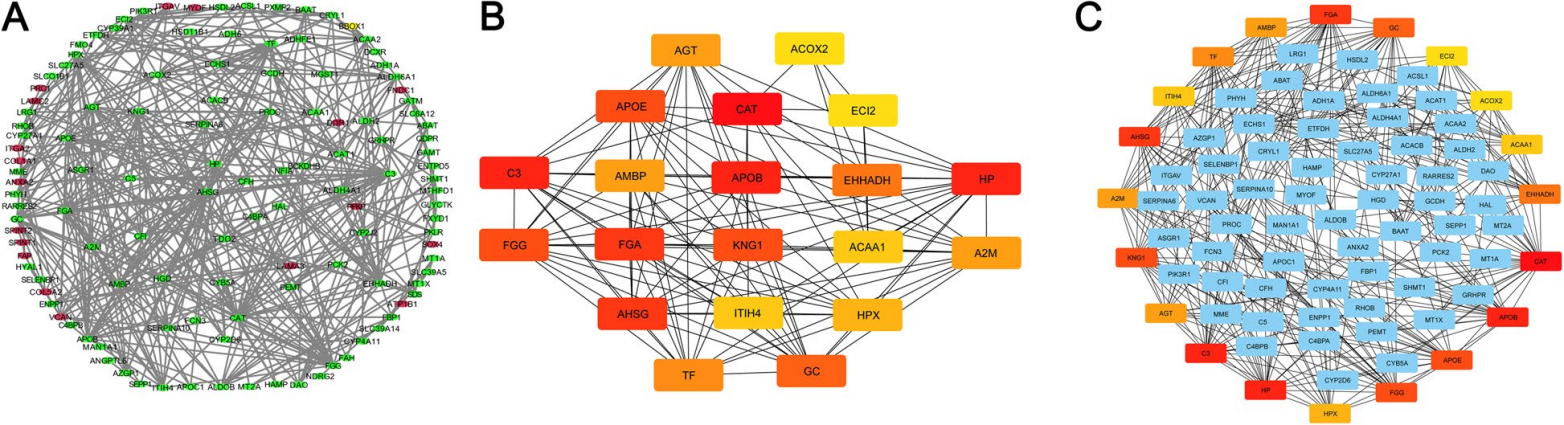
PPI networks of DEGs. **A** Upregulated and downregulated genes in PPI networks (Red nodes mean upregulated genes, and green nodes mean downregulated genes). **B** Hub genes in PPI networks. **C** Hub genes and other genes in PPI networks

We used The Search Tool for the Retrieval of Interacting Networks Genes (STRING) to draw a protein–protein interaction (PPI) network and the network was modified by cytoscape software 3.8.2. There were 146 nodes and 464 edges comprised in the PPI network (Fig. 6). Then, we calculated the degree of connectivity and screened the top 20 genes in the PPI network using cytoHubba App (Table 4). All the twenty hub genes were downregulated in ICC compared with that in normal liver tissue. The rank of the twenty hub genes were listed in the Table 4. After that, we used DAVID tools to make the functional analyses of hub genes (Table 5). The results of GO analysis showed that hub genes were mainly enriched in CCs, including blood microparticle, extracellular region, extracellular space, extracellular exosome, peroxisomal matrix, platelet alpha granule lumen and intracellular membrane-bounded organelle. BP analysis indicated that the hub genes were enriched in platelet degranulation, negative regulation of endopeptidase activity and receptor-mediated endocytosis. And for the MF, the hub genes were enriched in receptor binding. Besides, the results of KEGG analysis indicated that hub genes were enriched in complement and coagulation cascades and peroxisome.

**Table 4:** Rank of the top 20 genes in the PPI network

Rank	Gene symbol	Gene description	Degree
1	CAT	Catalase	28
2	APOB	Apolipoprotein B	24
2	HP	Aaptoglobin	24
2	C3	Complement component 3	24
5	FGA	Fibrinogen alpha chain	23
5	AHSG	α2-HS-glycoprotein	23
7	APOE	Apolipoprotein E	22
7	KNG1	Kininogen 1	22
7	FGG	Fibrinogen gamma chain	22
10	GC	Group-specific component (vitamin D binding protein)	21
11	EHHADH	Enoyl-CoA, hydratase/3-hydroxyacyl CoA dehydrogenase	19
12	TF	Transferrin	18
13	A2M	α2-macroglobulin	17
13	AMBP	Alpha-1-microglobulin	17
13	AGT	Angiotensinogen (serpin peptidase inhibitor, clade A, member 8)	17
16	HPX	Hemopexin	16
17	ITIH4	Inter-alpha-trypsin inhibitor heavy chain family, member 4	15
17	ACAA1	Acetyl-CoA acyltransferase 1	15
19	ACOX2	Acyl-CoA oxidase 2, branched chain	14
19	ECI2	Enoyl-CoA delta isomerase 2	14

**Table 5:** Functional analyses of hub genes

Category	Term	Description	Count	FDR
BP term	GO:0002576	Platelet degranulation	7	3.68E-07
BP term	GO:0010951	Negative regulation of endopeptidase activity	7	4.89E-07
BP term	GO:0006898	Receptor-mediated endocytosis	5	0.003905848
CC term	GO:0072562	Blood microparticle	14	7.69E-22
CC term	GO:0005576	Extracellular region	15	3.37E-10
CC term	GO:0005615	Extracellular space	14	5.85E-10
CC term	GO:0070062	Extracellular exosome	16	1.78E-08
CC term	GO:0005782	Peroxisomal matrix	5	1.42E-06
CC term	GO:0031093	Platelet alpha granule lumen	5	2.46E-06
CC term	GO:0043231	Intracellular membrane-bounded organelle	6	0.001267314
MF term	GO:0005102	Receptor binding	9	1.63E-07
KEGG pathway	hsa04610	Complement and coagulation cascades	5	2.29E-04
KEGG pathway	hsa04146	Peroxisome	5	2.40E-04

### Survival analysis of twenty hub genes

**Fig. 7:**
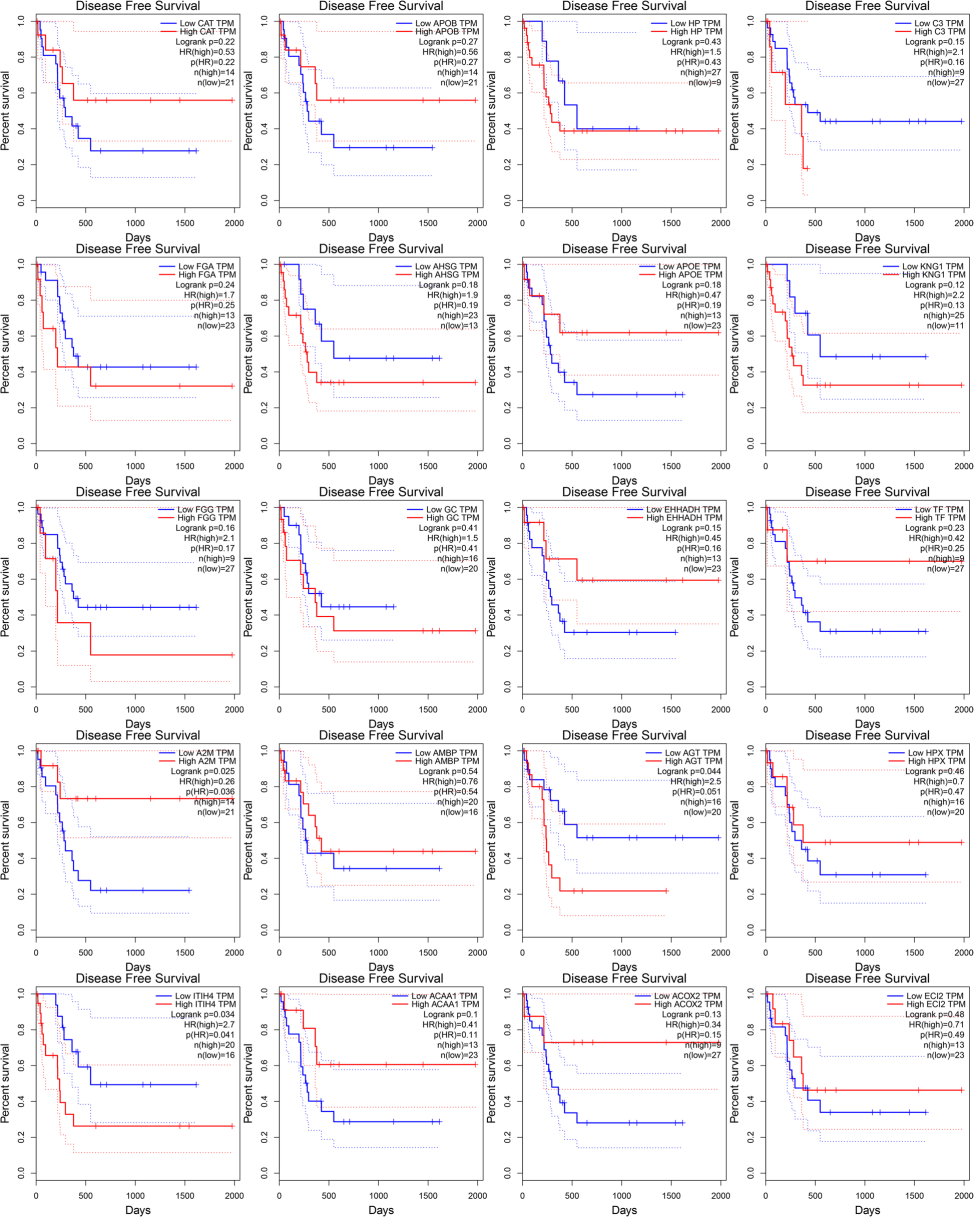
Disease free survival analyses of the top twenty hub genes in ICC patients. Abbreviations: CAT, catalase; APOB, apolipoprotein B; HP, haptoglobin; C3, complement component 3; FGA, fibrinogen alpha chain; AHSG, α2-HS-glycoprotein; APOE, apolipoprotein E; KNG1, kininogen 1; FGG, fibrinogen gamma chain; GC, group-specific component (vitamin D binding protein); EHHADH, enoyl-CoA, hydratase/3-hydroxyacyl CoA dehydrogenase; TF, transferrin; A2M, α2-macroglobulin; AMBP, alpha-1-microglobulin; AGT, angiotensinogen (serpin peptidase inhibitor, clade A, member 8); HPX, hemopexin; ITIH4, inter-alpha-trypsin inhibitor heavy chain family, member 4; ACAA1, acetyl-CoA acyltransferase 1; ACOX2, acyl-CoA oxidase 2, branched chain; ECI2, enoyl-CoA delta isomerase 2

The Gene Expression Profiling Interactive Analysis (GEPIA) tool was used to determine whether the twenty potential key genes had the prognostic values. Data of only 36 ICC patients were available for the analysis of disease free survival analysis. The best cutoff value was selected manually. *P* < 0.05 was supposed to be statistically significant, and three of the twenty hub genes were prognostic markers for the disease free survival analysis in ICC patients, but the survival analysis results of AGT and ITIH4 were contradictory to our previous analysis, that was the DEGs analysis results showed that they were downregulated in ICC compared with that in normal bile duct tissue, while the survival analysis showed they were favorable for the ICC patients when they were downregulated. Only A2M was the potential prognostic gene for disease free survival analysis in ICC patients (Fig. 7).

## Discussion

Carcinoma of bile duct is divided into ICC and extrahepatic cholangiocarcinoma. According to the general type of tumor, ICC is divided into mass type, periductal infiltration type and intraductal growth type, and its incidence is second only to hepatocellular carcinoma in the primary liver cancer. In the past 20 years, the incidence of ICC has been on the rise globally [12, 13]. At present, the main indicator of primary liver cancer (PLC) in clinical diagnosis is alpha fetoprotein (AFP), which has been widely used in the general survey and screening of high-risk groups [14]. In China, 30 to 40% of PLC patients are negative for AFP, while ICC patients are almost all negative for AFP [15]. Most ICC patients are in advanced stage when they are diagnosed due to the lack of specific early symptoms of cholangiocarcinoma and negative AFP. ICC is not sensitive to radiotherapy and chemotherapy, and lacks effective targeted drugs [16]. Currently, medical therapy can not significantly improve the therapeutic effect and prolonged the survival period of patients [17, 18]. Radical resection is the only chance of long-term survival for ICC patients, but the tumor is locally invasive and often metastasizes, especially through the lymphatic system. Because of lacking prognostic marker, the prognosis of ICC patients is still very poor even after surgical resection [19–22]. Most ICC patients have lost the opportunity of radical resection since the diagnosis is too late [23]. For patients with ICC, the lack of early diagnostic index and prognostic indicators, poor efficacy of various treatment methods lead to poor clinical outcome. Hence, it is urgent to explore new diagnostic, therapeutic and prognostic targets of ICC.

The determination of the molecular mechanism about tumorigenesis is very important for the diagnosis and treatment of cancer patients [24]. There is an urgent need to study the pathogenesis of intrahepatic cholangiocarcinoma and the knowledge gained may help us to develop new clinical treatment strategies [25]. In recent years, high-throughput sequencing technology and bioinformatics analysis have been increasingly applied to biological research [26]. Bioinformatics is an interdisciplinary discipline, which uses bioinformatics methods to dig out data at the molecular level, and provides a new way for studying the molecular mechanism of various diseases [27]. Large amounts of data are stored in several common databases such as GEO (https://www.ncbi.nlm.nih.gov/geo/) ICGC (https://dcc.icgc.org/) and ENCODE (https://www.encodeproject.org/) [28]. Compared with a single high throughput screening dataset, integrating some high throughput screening datasets (RNA sequencing and cDNA microarray) is regarded as a better way to improve the reliability of results [29–31]. In this study, an in-silico analysis was performed using bioinformatics methods to screen and identify new molecular targets. We firstly obtained DEGS by comparing ICC tissues with normal samples based on five microarray datasets that were selected by the comparison program from the GEO database. A total of 28 upregulated DEGs and 118 downregulated DEGs were identified and we performed GO and KEGG pathway enrichment analysis of DEGs. The downregulated genes were mainly enriched in BPs and CCs, including oxidation-reduction process, negative regulation of endopeptidase activity, extracellular exosome, metabolic process and so on, and significantly concentrated in the KEGG terms metabolic pathways, fatty acid degradation, biosynthesis of antibiotics, complement and coagulation cascades, peroxisome and carbon metabolism. The upregulated genes were principally concentrated in extracellular matrix organization, cell adhesion, ECM-receptor interaction, focal adhesion and PI3K-Akt signaling pathway. Then, a PPI network was built to research the correlation of the DEGs, and twenty hub genes that were all downregulated in ICC were discovered by cytoscape 3.8.2, including CAT, APOB, HP, C3, FGA, AHSG, APOE, KNG1, FGG, GC, EHHADH, TF, A2M, AMBP, AGT, HPX, ITIH4, ACAA1, ACOX2 and ECI2. Finally, we used The Gene Expression Profiling Interactive Analysis (GEPIA) online tool to explore the relationship between hub genes and prognosis of ICC patients. Based on the results, overexpression of A2M was related to favorable prognosis of ICC patients. Therefore, overexpression of A2M might be a favorable prognostic factor of ICC patients.

A2M, also known as α2-macroglobulin, is a core modulator in controlling protease activity and cell proliferation. α2-macroglobulin acts as protease inhibitor, hormone, immune modulator and cytokine [32] and some research has demonstrated that α2-macroglobulin as a kind of macromolecular plasma protein in the blood, α2-macroglobulin can inactivate a variety of proteases by inhibiting plasmin and kallikrein [33, 34], and can also act as the carrier protein that binds to growth factors, hormones, and cytokines such as platelet derived growth factor (PDGF), basic fibroblast growth factor (bFGF), insulin like growth factor (IGF) and interleukin [35]. There is evidence that A2M can affect TGF-β1 and other growth regulator ligands after binding to its receptor LRP1 [36]. Fears CY et al. showed that the combination of α2-macroglobulin and LRP1 also phagocytosed a variety of matrix metalloproteinases, such as MMP-2, thereby inhibiting the migration and invasion of tumor cells [37].

In addition, α2-macroglobulin is closely related to Alzheimer’s disease. Alzheimer’s disease (AD) is the most familiar neurodegenerative disease among the elderly people. α2 macroglobulin that can be synthesized by astrocytes and neurons in the brain is a high affinity binding protein of amyloid β protein (Aβ), and its 27 amino acids at C-terminal specifically bind with Aβ peptide to neutrinate Aβ toxicity [38, 39]. A2M can be divided into 6 fragments by restriction enzyme digestion and PCR methods, namely FP1 (aa99-392), FP2 (aa341-590), FP3 (aa591-744), FP4 (aa775-1059), FP5 (aa1030-1279) and FP6 (aa1242-1451). Aβ binds to FP6 segment with high specificity [40], suggesting that FP6 may become a new direction for the treatment of Alzheimer’s disease [41].

Birkenmeier et al. showed that the decline of blood A2M in the elderly people was highly correlated with the incidence of tumors [42] and Lindner et al. reported that A2M binded with its receptor LRP1 to inhibit the Wnt/β-catenin tumor signaling pathway [43]. Lauer et al. believed that A2M combined with growth factors to inactivate known tumor growth factors, thereby inhibiting tumor growth and invasion [44]. Wood et al. revealed the significance of A2M in the regulation of clock genes [45]. The mechanism of the relation between the circadian clock and cancer is not clear, but destruction of circadian rhythm is associated with tumorigenesis [46]. There is evidence that A2M regulates tumor cell growth by upregulating PTEN and inhibiting tumour promoting signalling pathways such as PI3K/AKT, SMAD, and A2M is likely to become a new type of therapeutic drug [47]. Increasing the proportion of activated A2M in vivo has been considered for use in the treatment of cancer [48].

In short, the relationship between A2M and ICC has not been fully understood, and it is necessary to further explore the molecular mechanism of A2M and ICC. However, the anticancer effect of A2M suggests that overexpression of A2M might be associated with the better prognosis in patients with ICC, and in our study, the expression of A2M was low in ICC which was closely related to the poor prognosis of ICC. A2M is highly likely to be a therapeutic target for ICC.

## Conclusion

We found 146 DEGs containing 28 upregulated genes and 118 downregulated genes between ICC and normal bile duct tissues based on the selected five datasets from the GEO database. Among them, A2M was the potential core gene of ICC. Overexpression of A2M was closely related to better prognosis in ICC patients. The results of our study need further research to confirm. In conclusion, A2M might be a potential target for the treatment of ICC.

## Data Availability

The raw data of this study are freely available from the website https://www.ncbi.nlm.nih.gov/geo, and all the analyzed data are included in this manuscript.
